# New burn model for developing consistent second- and third-degree burn injuries in rats

**DOI:** 10.1186/s13104-025-07200-8

**Published:** 2025-04-16

**Authors:** Ahmed Ibrahim, Khaled M. A. Hassanein, Mahmoud Soliman, Abdelnaby M. Elshahawy

**Affiliations:** 1 Veterinary Teaching Hospital, Faculty of Veterinary Medicine, Assiut University, Assiut, 71526 Egypt; 2 Department of Pathology and Clinical Pathology, Faculty of Veterinary Medicine, Assiut University, Assiut, 71526 Egypt; 3 Department of Physics, Faculty of Science, Assiut University, Assiut, 71516 Egypt

**Keywords:** Burn, Drying oven, Model, Rat, Stainless steel

## Abstract

**Objective:**

This study’s aim was: (1) introduce the digital drying oven as a reproducible, controllable, and accurate heating device for burn model creation. (2) Define the heating temperature appropriate for developing consistent second and third-degree burn injuries in rats.

**Results:**

Burns appeared deeper with more distinct borders in groups (B) and (C) than in group (A). The stainless-steel rod at 100 ºC created burn injuries of the second degree, evidenced by the sloughing of the epidermis and necrosis in the epithelium and upper part of the dermis. Heating at 150 and 200 ºC created third-degree burn injuries, where necrosis involved the epidermis and dermis and extended to the subcutaneous fat and muscles. The depth of the burn wound in the group (B) (371.2 ± 41.3 μm) and (C) (385.2 ± 38.0 μm) was significantly deeper compared with the group (A) (178 ± 46.6 μm) (*P* < 0.001). The digital drying oven is a reliable, reproducible, and controllable heating device for creating burn models. The stainless-steel rod (63 g and 8 mm) heated at 100 and 150 ºC with a contact time of 30 s is adequate for creating consistent second and third-degree burn injuries in rats, respectively.

## Introduction

Burn injuries remain a major health concern as they can lead to high mortality and morbidity rates. Burns can be thermal, chemical, electrical, or radiation according to the causative object. However, thermal burn represents the most common type of burn injury, making up about 86% of burned patients. It is caused by contact with hot surfaces, hot liquids, steam, or flame [1]. Burns are classified into four types according to the depth of injury. First-degree or superficial, in which only the epidermis is involved. Second-degree or partial thickness, in which the papillary dermis (superficial partial thickness) or reticular dermis (deep partial thickness) are involved. Third-degree or full thickness, in which the epidermis and dermis are involved, and the injury extends to the subcutaneous layer (hypodermis). Deeper structures, including the muscles, tendons, ligaments, and bones are involved in the fourth-degree burn [1, 2]. However, burns are dynamic, and burn depth can progress to being deeper [2].

The treatment and management strategies of burns vary according to the depth of injury [2]. Therefore, the layers of the skin involved in burn models should replicate the clinical picture of burn degrees to maximize the usefulness and accuracy of these models [3]. Various experimental animal models have been designed to study the pathophysiology of burns and evaluate the healing efficacy of several materials on different burn degrees [3, 4]. Among the different animal species, rats are the most used animal models for thermal burns [5]. The hot water model has been extensively used and considered the standard burn model in rats. However, this model has been described in various studies with diversity in temperature, contact time, weight, material, diameter, and shape of the metal device, indicating a lack of standardization and uniformity of the developed burn degrees [4, 6–8]. For instance, a heated brass bar in boiling water was reported for the conduction of third-degree burns [9, 10] and second-degree burns [11] under the same criteria in a rat model. Standardization of the metal device material, diameter, shape, weight, temperature, and contact time in a burn model is required to create a consistent burn degree.

The hypothesis was that using the digital drying oven to heat a metal device at a controlled digital temperature would be more accurate, reliable, and convenient than heating in boiling water, allowing the chance to test higher temperatures for burn model development. Therefore, this study’s aim was: (1) introduce the digital drying oven as a reproducible, controllable, and accurate heating device for burn model creation. (2) Define the heating temperature appropriate for developing consistent second and third-degree burn injuries in rats.

## Methods

### Experimental animals

This protocol was approved by The Research Ethics Committee (REC) of the Faculty of Veterinary Medicine, Assiut University, Assiut, Egypt, and conducted in compliance with the ARRIVE guidelines and the relevant guidelines and regulations for the care and use of laboratory animals in research and education. The study was conducted on fifteen adult Wistar-Albino rats (*n* = 15), obtained from the Experimental Animal House, Faculty of Veterinary Medicine, Assiut University, Assiut, Egypt, weighing 190–200 g (g). Rats were housed in individual cages with *ad libitum* access to feed and water in an air-conditioned room (22–24 ºC, 55–56% humidity) with a 12-h light and 12-h dark cycle. Rats were randomly allocated to three experimental groups of five rats each (*n* = 5) as described below.

### Conduction of burn injuries

**Fig. 1:**
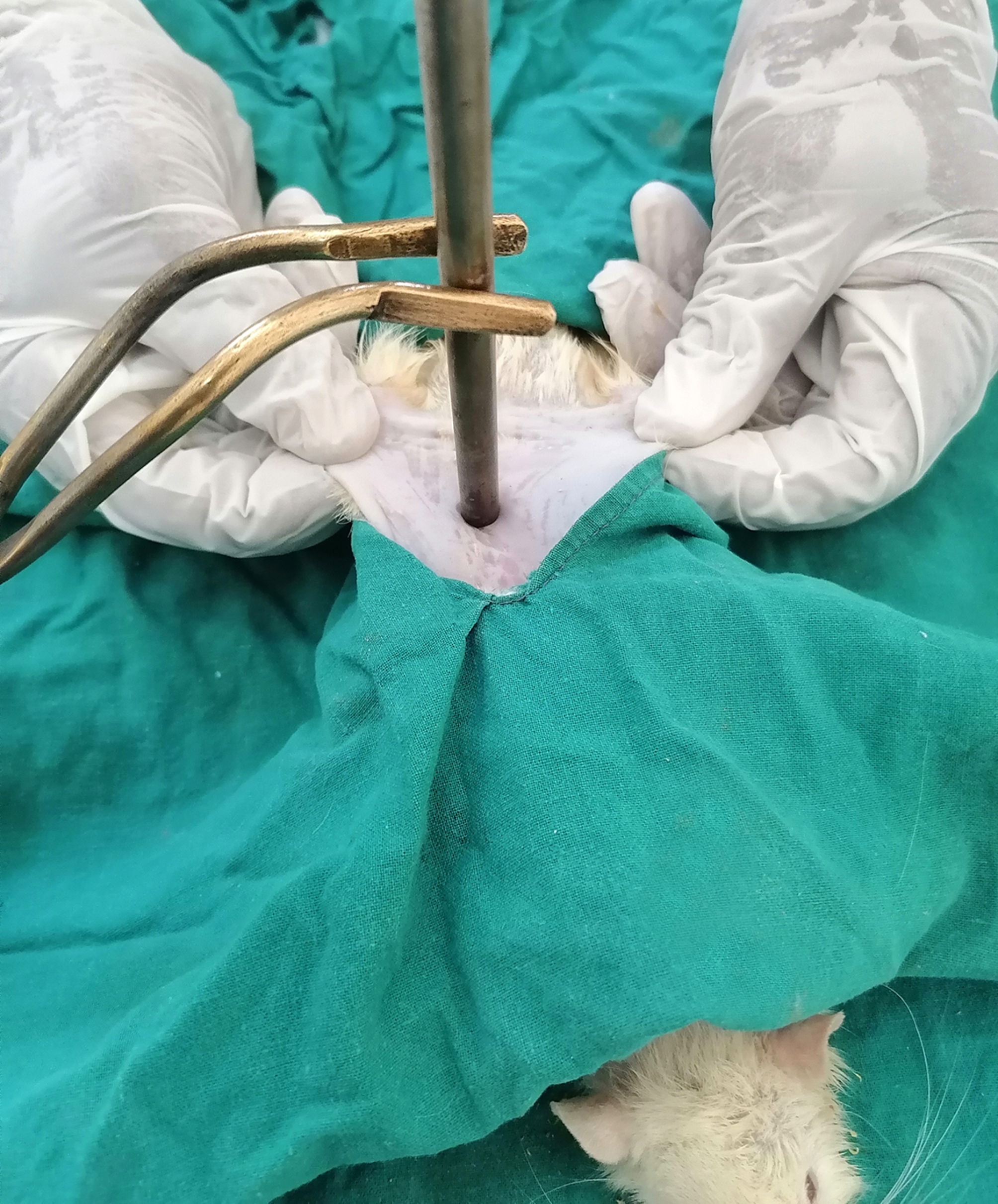
The dorsum skin of the rat pulled away from the underlying, creating a flat surface. The heated rod was held by a tong and maintained to rest the skin on its own weight

Burn injuries were conducted under the effect of general anesthesia using 2% xylazine HCl (Xyla-Ject, ADWIA Co., SAE, Egypt) at a dose of 1 mg/100 g and 5% ketamine HCl (Ketamine, Sigma-tec Pharmaceutical Industries, SAE, Egypt) at a dose of 4 mg/100 g intramuscularly (IM) in one syringe [12]. The dorsum of rats was shaved and disinfected with 10% povidone-iodine solution (BETADINE, El- Nile Co. for Pharmaceutical and Chemical Industries, Egypt). A stainless-steel rod weighing 63 g and 8 mm in diameter was heated at 100, 150, and 200 ºC in the digital drying oven (DHG-9075 A, with a maximum temperature of 300 ^o^C) and then maintained for 30 min in the oven at the pre-determined temperature before being used for conducting burn injuries in groups (A), (B), and (C), respectively. A toe-pinch test was applied to confirm the efficiency of anesthesia immediately before burning. The skin was elevated away from the underlying tissue, creating a flat surface. The heated rod was held by a tong and maintained to rest the skin on its weight with a contact time of 30 s in all rats [13] (Fig. 1). All burns were inflicted near the digital drying oven (10–20 cm). Burns were digitally photographed and underwent a macroscopic and microscopic examination by a pathologist who was blinded to the tested groups.

### Macroscopical examination

Burn injuries were examined for uniformity, even surfaces, color, edges, and mechanical injury (detached epithelium).

### Microscopical examination

Tissue specimens of burn wounds were carefully dissected and fixed in 10% neutral buffered formalin. The formalin-fixed samples were routinely processed (dehydrated in graded alcohol series, cleared in xylene, embedded in paraffin, and sectioned). Serial 4 μm sections were stained with Mayer’s hematoxylin (Merck, Darmstadt, Germany) and eosin (Sigma, Missouri, USA). The slides were then examined microscopically by an Olympus CX31 microscope and photos were taken by an Olympus C-5060 camera adapted into the microscope. The condition of the epidermis, dermis, hypodermis (subcutaneous), and skeletal muscle were evaluated. The depth of the burn wounds (µm) (5 images/group) was measured using ImageJ software (National Institutes of Health, Bethesda, MD, USA).

The anesthetized animals were humanely euthanized physically with cervical dislocation by a proficient person according to the AVMA guidelines for the euthanasia of animals [14]. The thumb and index finger are placed on either side of the neck at the base of the skull. A rod is pressed at the base of the skull. With the other hand, the base of the tail or the hind limbs is quickly pulled, causing separation of the cervical vertebrae from the skull. Death was confirmed (absence of breathing and heartbeats) before disposal of the animals (buried deeply according to local laws and regulations).

### Statistical analysis

Data are presented as means ± standard deviation. The statistical analysis used GraphPad Prism software version 8.0.1 (GraphPad Software Inc., La Jolla, CA, USA). One-way analysis of variance (ANOVA) followed by Tukey’s post hoc test was used to analyze the depth of the burn wound. A *p*-value < 0.05 was considered statistically significant.

## Results

### Macroscopic examination

**Fig. 2:**
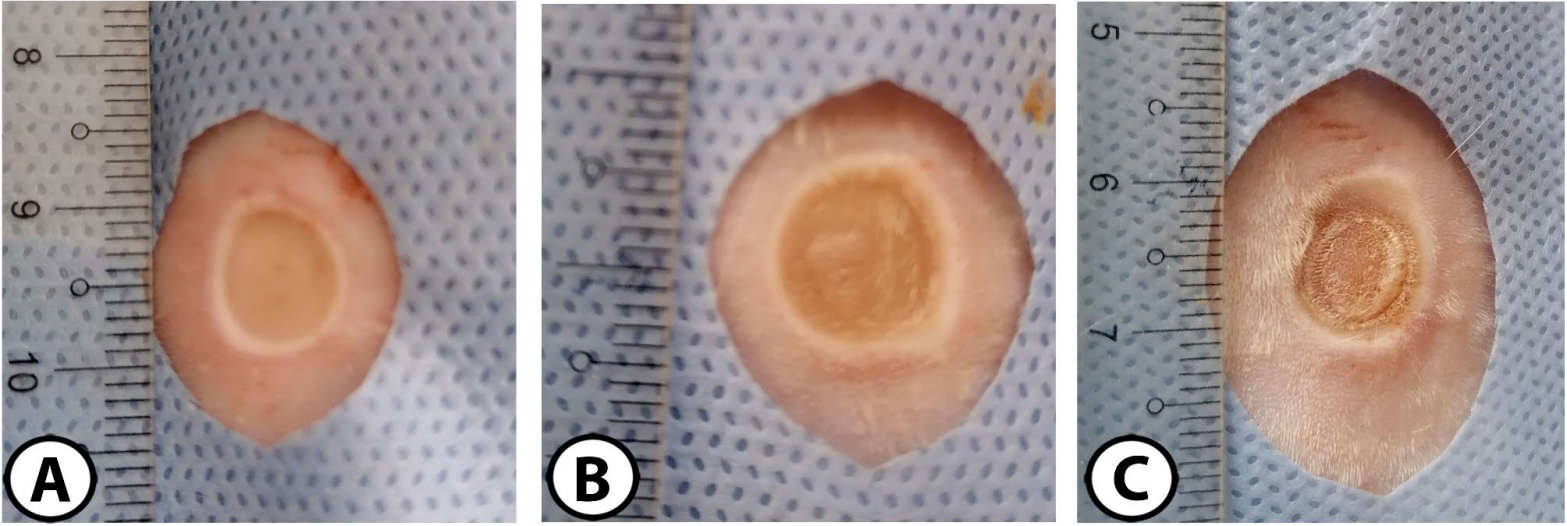
Gross appearance of burn injuries of second (**A**) and third-degree (**B** & **C**) in a rat model

The burn injuries were uniformly round in all rats with distinct lines of demarcation and even surfaces. Burns were pale in group (A), brownish in group (B), and chalky white in group (C). Burns appeared deeper with more distinct borders in groups (B) and (C) than in group (A). There was no evidence of any mechanical injury (epithelium detachment) in all burn wounds (Fig. 2).

### Microscopic examination

**Fig. 3:**
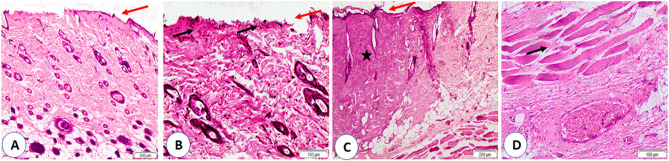
Group (**A**) showed second-degree burns with sloughing of the epidermis (red arrows) (**A**) and necrosis of the upper dermis (black arrow) (**B**). Groups (**B** & **C**) showed third-degree burns with sloughing of the epidermis (red arrows) and necrosis of the dermis that extended to the deep layers (stars) (**C**) and muscles (black arrows) (**D**). H&E

**Fig. 4:**
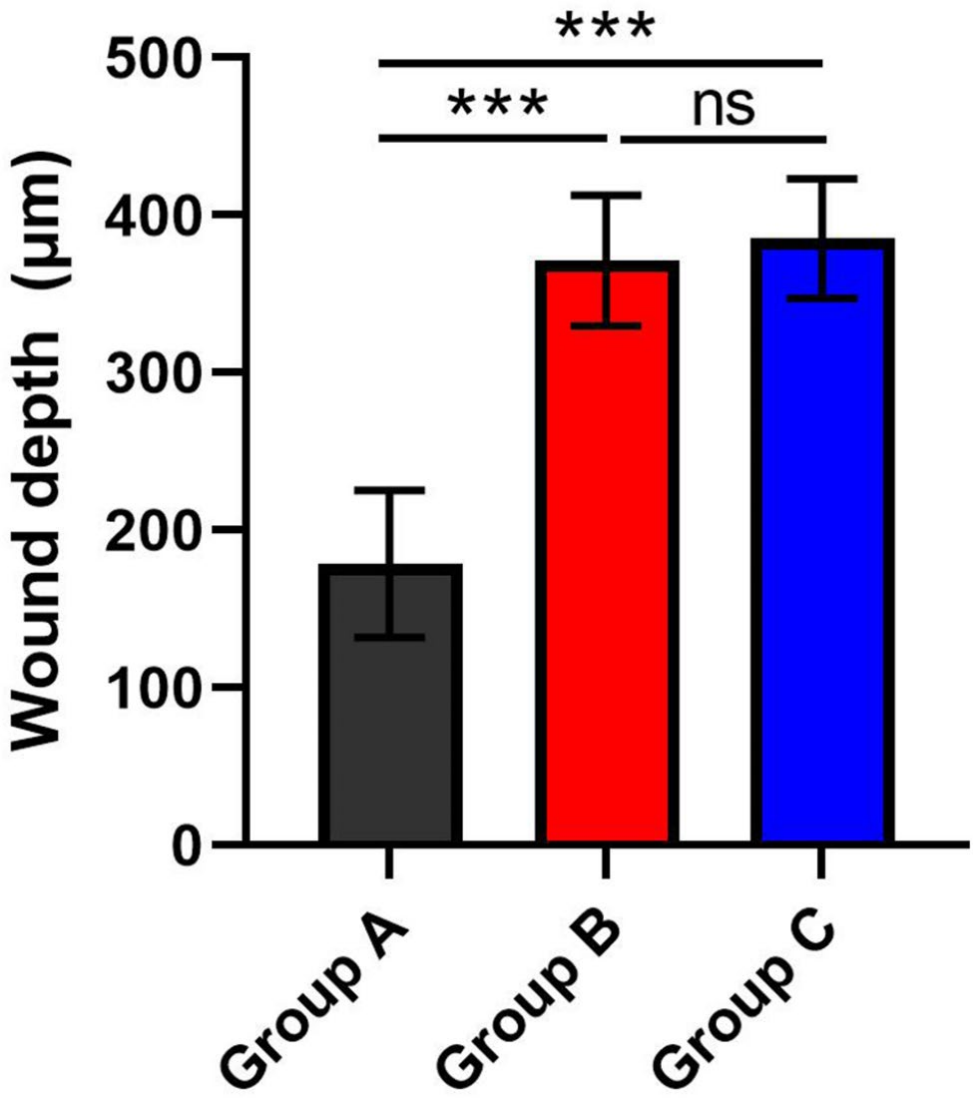
Burn wound depth (µm) in **A** (178.4 ± 46.6), **B** (371.2 ± 41.3), and **C** (385.2 ± 38.0) groups. ***p < 0.001

Histopathological examination of the burn wounds in the study groups revealed second and third-degree burns. A stainless-steel rod heated at 100 ºC in group (A) created burn injuries of the second degree. There was a separation of the epidermis from the dermis with evidence of necrosis in the epithelium and upper part of the dermis. The skin adnexa and deep dermis showed a normal appearance (Fig. 3A & B). The heating of the rod at 150 and 200 ºC created burn injuries of the third degree in groups (B) and (C), respectively, in which necrosis involved the epidermis and dermis and extended to the subcutaneous fat and muscles (Fig. 3C & D). In addition, the depth of the burn injury in the group (B) (371.2 ± 41.3 μm) and (C) (385.2 ± 38.0 μm) was significantly greater than that in the group (A) (178 ± 46.6 μm) (*P* < 0.001) (Fig. 4).

## Discussion

This study introduced the digital drying oven as a new, reliable, reproducible, and controllable heating device for developing standardized burn models in rats. Moreover, the study defined the standard temperature (100 and 150 ºC) and contact time (30 s) required for developing consistent second and third-degree burn injuries using a stainless-steel rod (63 g and 8 mm), respectively.

The main objective of the development of burn models is to simulate the injured layers of skin in different burn degrees under defined temperatures and exposure times [4]. Animal models of burn as opposed to in vitro models can capture post-burn pathophysiology and clinical features of burn injury [3]. Rats are the most frequently used species in burn models. This is because of their size, easy maintenance, and transgene generation in laboratory settings. In contrast, larger animal models such as rabbits and pigs require higher costs and extended experimental time [5]. Although rats share humans several physiological and pathological features, rat models for burns are not the most similar to human burn injuries [5]. This is due to the higher skin elasticity and thinner epidermis and dermis than humans [15, 16]. However, rat models are still essential for understanding burn’s molecular and cellular aspects [17].

Different methods have been used for heating the metal devices for burn infliction in rat models, including hot water, open flame, electricity, and boiling oil [18–22]. However, boiling water is the most used method [8, 10, 11, 16]. The temperature of the heated devices varied from 60 to 100 ºC [10, 23]. Some studies failed to monitor the temperature of the heated devices [18, 24], while others developed methods for monitoring the temperature of the heated device including the use of a digitalized multimeter [25] and a thermocouple [16]. Moreover, wiping the heated device from the water before application on the skin may cause a degree of heat loss.

Here, using the digital drying oven to heat the metal rod represents a convenient, controllable, and reproducible method compared to others of maintaining an accurate temperature of the used rod for burn infliction. Moreover, the heated rod was maintained in the drying oven for 30 min after reaching the pre-determined temperature for ensuring heat stabilization.

Metals made of brass [10, 11], aluminum [26], iron [7], and stainless steel [16] have been used in previous studies. However, other studies ignored the type of metal material [18, 25]. The stainless-steel rod was used in this study as the thermal conductivity of stainless steel is low (16 WmK), compared to aluminum (225 WmK) or brass (109 WmK). This prevents heat loss from the heated stainless-steel rod and preserves its temperature to a great extent during burn infliction [16, 27]. Although the weight of the metal device (30–51 g) and duration of contact (2–40 s) [28, 29] varied between studies, they are important determinants of burn depth [16]. Maintenance of the heated rod to rest perpendicular to the skin on its weight without applying any pressure from the operator’s hand ensures a homogenous burn depth [10, 16]. In addition, upward pulling of the skin away from the underlying tissues is essential to avoid irregular dorsum surfaces providing burn wounds with consistent depth. The burn was induced while the rats were near (10–20 cm) the drying oven to lessen the heat loss from the heated rod [16].

The heating of the stainless-steel rod at 100 ºC created a second-degree burn injury. This was confirmed by the microscopic examination, where there was sloughing to the epidermis and necrosis in the epithelium and upper part of the dermis. Third-degree burn injuries were developed at 150 and 200 ºC. Necrosis involved the epidermis and dermis and extended to the subcutaneous fat and muscles [30, 31].

### Limitations

Further studies are still needed to compare hot water versus the drying oven as methods for probe heating, other than testing different metal probes on a larger sample size. Also, burns can progress over time, especially from second- to third-degree. This study does not assess burn wound progression or healing. Therefore, the progress of burn injuries needs to be addressed in the future.

## Data Availability

All data generated or analyzed during this study are included in this published article.
